# ARM’s Perspective on the First Joint Clinical Assessments for ATMPs: Challenges and Opportunities on the Path Ahead

**DOI:** 10.3390/jmahp13020014

**Published:** 2025-04-03

**Authors:** Paolo Morgese, Stephen Majors, Dilip Patel

**Affiliations:** 1 Alliance for Regenerative Medicine (ARM), B-1040 Brussels, Belgium; smajors@alliancerm.org; 2 Autolus Therapeutics PLC London, London W12 7FP, UK; d.patel@autolus.com

**Keywords:** HTA Regulation, Joint Clinical Assessment, HTA, ATMPs

## Abstract

Advanced Therapy Medicinal Products (ATMPs) are revolutionising modern medicine. By addressing the root cause rather than the symptoms of disease, ATMPs hold the promise of long-lasting benefits or even cures for severe, genetic, and rare diseases—including rare cancers—for patients with few or no viable treatment options. At the same time, the inherent complexities of ATMPs pose challenges to traditional HTA frameworks. Unlike conventional treatments, ATMPs are often one-time therapies with a high magnitude of effect. However, their long-term durability remains uncertain at launch. The Joint Clinical Assessment (JCA), under the EU’s Health Technology Assessment (HTA) Regulation, represents a once-in-a-generation opportunity to consolidate the strengths of national HTA processes into a unified framework that accounts for the specificities of ATMPs and streamlines decision-making, cementing Europe’s position as a pioneer in innovative HTA approaches. While concerns remain regarding the suitability of current JCA methodologies for ATMPs, the HTA Regulation continues to bring the HTA ecosystem closer together, with numerous benefits already emerging from EU-wide collaboration on JCAs. This article outlines the HTA challenges posed for and by ATMPs, and ARM’s perspective on the JCA’s implementation. A ‘fit for purpose’ JCA holds the promise to unlocking these therapies’ benefits for individuals across Europe.

## 1. Introduction—The Revolutionary Potential of ATMPs

Advanced Therapy Medicinal Products (ATMPs) are revolutionising modern medicine. In the European Union (EU), ATMPs are defined as medicines for human use that are based on genes, tissues, or cells [1,2,3]. By addressing the root cause, rather than the symptoms of disease, they hold the promise of long-lasting benefits or possibly even cures, transforming the treatment landscape for severe, genetic, and rare diseases—including rare cancers—and offering new hope to patients who often have few or no viable treatment options. Indeed, the average life expectancy for a rare genetic disease with an approved gene therapy is below 40 years [4,5], indicating the high unmet medical need that ATMPs address.

When standards of care are available, they are often very costly. The lifetime standard of care for some these diseases can run to the millions of Euros. For example, the annual cost of treating patients with severe haemophilia has been estimated in France, Germany, Italy, and Spain at EUR 173,102; EUR 313,068; EUR 210,235; and EUR 132,329, respectively, resulting in lifetime costs that can run well into the millions of Euros [6]. As many ATMPs require only a single administration, ATMPs have the potential to save healthcare systems money in the long term, while at the same time significantly reducing the burden of treatment for patients.

The potential for ATMPs is already being realised with cell-based therapies such as CAR-Ts for fast-progressing cancers [5], and gene therapies for severe genetic and rare diseases such as spinal muscular atrophy (SMA), metachromatic leukodystrophy (MLD), and sickle cell disease. The anticipated broadening of applications of ATMPs to more common diseases such as diabetes, autoimmune diseases, Parkinson’s, and cardiovascular disease will further transform the healthcare landscape and improve patient outcomes [5,7].

The Alliance for Regenerative Medicine (ARM) is the leading international advocacy organisation championing the benefits of engineered cell therapies and genetic medicines for patients, healthcare systems, and society. ARM represents over 400 members across 25 countries, including emerging and established biotechnology companies, academic and medical research institutions, and patient organisations. As a community, ARM builds the future of medicine by convening the sector, facilitating influential exchanges on policies and practises, and advancing discussions through data and analysis. As the global voice of the sector, ARM actively engages key stakeholders to enable the development of advanced therapies and to modernise healthcare systems so that patients benefit from durable, potentially curative treatments [8].

As part of our work, we have closely followed the entry into force and implementation of the Health Technology Assessment (HTA) Regulation [9]. We are a member of the EU’s HTA Stakeholder Network—set up to facilitate dialogue between relevant stakeholder organisations (including patient associations, non-governmental organisations in the field of health, health technology developers (HTDs), and health professionals) along with the HTA Coordination Group.

This article sets out our view of the process so far, with particular regard to the specific challenges relating to HTA methods when assessing ATMPs and the promise the Joint Clinical Assessment holds for EU patients with genetic diseases, cancers, and other high-unmet-need conditions addressed by ATMPs. Looking ahead to the first JCAs in 2025, we set out our recommendations for the success of a JCA that delivers for patients in Europe.

## 2. The Promise of the Joint Clinical Assessment for ATMPs

The EU’s Health Technology Assessment (HTA) Regulation [10], adopted in December 2021, recognises a number of distinct problems with the current European HTA landscape. The introductory Recital to the Regulation specifically states that Member States’ (MSs) parallel assessments, and divergent processes and methodologies, can result in HTDs “being confronted with multiple and divergent requests for data” and “lead to both duplication and variation in outcomes” [9]. Indeed, “the duplication of submissions and consideration of different timings for submission across Member States can constitute a significant administrative burden for health technology developers, in particular for smaller companies with limited resources” [9].

The EU HTA Regulation is consciously designed to address some of these problems. By establishing the Joint Clinical Assessment (JCA), it proposes joint Member State cooperation in the clinical domains of the HTA process and, specifically, in the “identification of a health problem and current health technology, the examination of the technical characteristics of the heath technology under assessment, its relative safety, and its relative clinical effectiveness” [9]. By aiming to reduce the duplication of work linked to parallel national-level HTA processes, it attempts to speed up patient access to new therapies, improve predictability for companies on the processes and outcomes of clinical assessment, and strengthen the quality of HTA across member states [10,11].

That being said, the JCA report will not be legally binding for member states, and national HTA bodies still retain discretion to diverge from the report by conducting additional assessments or asking for further data. Importantly, the HTA Regulation explicitly states that value judgements on clinical benefit and relative effectiveness cannot be made at the JCA level and are reserved for Member States [9]. As such, the clinical relevance or interpretation of the estimate of relative effectiveness and safety may differ between MSs when drawing conclusions regarding the clinical added value of a treatment at a national level [12]. Nevertheless, the JCA holds the promise of minimising the time and cost involved in bringing ATMPs to patients, potentially unlocking the benefits of these therapies for individuals across Europe.

Finally, the EU HTA Regulation, through the JCA, may over time also provide a robust framework in supporting cross-border patient pathways for ATMPs. With a limited number of patients and specialised treatment centres for severe genetic and rare diseases, cross-border healthcare (CBHC) is a critical part of patient access to cell and gene therapies. Today, the full burden of HTA submissions is on the health technology developer. A pan-European clinical assessment framework and a single JCA report could represent a useful platform for Member States in managing cross-border patient referrals and the development of real-world evidence (RWE) among specialised centres across the EU.

## 3. Is HTA a Challenge for ATMPs, or Are ATMPs Challenging for HTA?

Both perspectives hold true. HTA frameworks face significant difficulties in accommodating the unique characteristics of ATMPs, while the inherent complexities of ATMPs also test the adaptability of traditional HTA methodologies.

For a majority of ATMPs, HTA processes and methods can be particularly challenging. HTA methods were initially developed and designed to assess and compare new conventional medicines to existing treatments, typically using data from randomised controlled trials (RCTs) to demonstrate their relative effectiveness. Innovative conventional medicines are typically administered continuously or according to a fixed schedule, addressing symptoms or, in some cases, modifying disease progression. These treatments often demonstrate a modest magnitude of effect, making well-powered randomised controlled trials (RCTs) with active comparators and one- to two-year follow-up periods an effective method for evaluating relative effectiveness and safety. These trial designs align well with the needs of both regulators and HTA bodies, which have traditionally focused on such short-term risk–benefit assessments [13]. ATMPs, however, challenge this framework. Unlike conventional treatments, ATMPs are often one-time therapies with a high magnitude of effect that are readily observable in the short term. Yet, what remains uncertain at the time of launch is the durability of their effect—whether these transformative treatments provide lifelong benefits or whether their impact diminishes over time. This inherent uncertainty complicates assessments of long-term relative effectiveness and safety, as seen recently with Tisagenlecleucel (a CAR-T therapy), where initial cost-effectiveness analyses underestimated its long-term value based on early trial data [14].

At the same time, ATMPs present distinct challenges for HTAs due to their fundamental differences from conventional medicines, particularly in clinical development, manufacturing, and assessment processes. ATMPs are often developed to target rare diseases or serious advanced-stage conditions, such as late-stage cancers, which inherently involve small, heterogenous patient populations. Of the 19 ATMPs with current EMA Marketing Authorisation, 17 target rare diseases or late-line cancer therapies with limited cohorts. This impacts what clinical trial designs are available. While RCTs remain the gold standard for evaluating the safety and efficacy of medical treatments and are used to assess ATMPs when possible [15,16,17], RCTs are often not feasible for ATMPs due to small patient populations and/or ethical concerns about placebo use when life-saving treatments might potentially be available, the absence of an effective standard of care, narrow treatment windows (for example, for progressive paediatric diseases), or the complexity and burden of treatment. As a result, 77% of ATMPs are licenced on the basis of non-RCT data, which inevitably introduces higher levels of uncertainty [18]. Where reliance on non-RCT data are necessary for ATMPs, uncertainty can be reduced by the use of established statistical methods, such as comparison to a propensity score-matched synthetic control arm, to mitigate potential bias [19].

While these challenges are well documented [20,21] and widely recognised [22], traditional HTA guidelines in Europe still frequently emphasise reducing uncertainty around long-term effects—an area often outside the scope of available evidence at launch—rather than focusing on the immediate and evident clinical benefits that are central to the value of ATMPs. That said, several national HTA bodies have developed specific pathways, adapted their methodologies, or taken pragmatic approaches during assessments, to ensure the potential value of ATMPs is assessed appropriately [18]. In particular, countries like the UK and Germany have changed their methodologies to simplify and accelerate the appraisal process, recognise disease severity, expand the development and use of robust RWE, and provide greater flexibility when managing uncertainty, while addressing and minimising the potential for bias [18]. In the same vein, the French *Haute Autorité de Santé* (Saint-Denis, France)has published new guidance on best practises for real-world evidence generation to support and assist the implementation of real-world studies for health products [18].

The reality is that traditional HTA frameworks have struggled to appropriately assess ATMPs, leading to delayed or restricted patient access. Highly effective therapies risk receiving poor HTA evaluations due to rigid and often unrealistic clinical trial requirements, undermining their potential to address high unmet medical needs. This misalignment both delays and limits access for patients and also places strain on the sector. While such evaluations do not always preclude positive pricing and reimbursement decisions, the reliance on non-adapted methods can lead to lengthier processes or sub-optimal decisions being made by payers due to perceived uncertainty.

The aim of flexibility in ATMP assessments is not to lower scientific standards, but to ensure that evaluation methodologies evolve in line with the unique characteristics of these therapies. This requires ongoing efforts to identify, develop, and refine robust methods that generate high-quality evidence while appropriately capturing the long-term value and transformative potential of ATMPs. It is possible to ensure greater flexibility in ATMP assessments without compromising patient safety or scientific integrity. Carefully designed observational studies, robust statistical adjustment techniques, and the use of external control arms can help mitigate bias and improve the reliability of evidence used in decision-making. By integrating these methodological safeguards, HTA frameworks can better capture the full value of ATMPs while maintaining high evidentiary standards.

## 4. A JCA ‘Fit for Purpose’: Adaptation for ATMPs and Setting Standards for RWE

The JCA represents a once-in-a-generation opportunity to consolidate the strengths of national HTA processes into a unified framework that accounts for the specificities of ATMPs. Ensuring a JCA that is ‘fit for purpose’ could contribute to decision-making processes that would reflect appropriately the value that ATMPs bring to patients [18,22]. By harmonising evidence standards and optimising the use of RWE to support clinical assessments, the JCA can reduce the redundancies and duplication of work, enhance the predictability for health technology developers (HTDs), and significantly accelerate patient access to ATMPs, particularly in more resource-scarce HTA settings.

Recognising the mutual challenges ATMPs and HTA frameworks present to one another (see above), the HTA Regulation explicitly calls for methodologies “adapted to include specificities of new health technologies for which some data may not be readily available. This may be the case for, inter alia, orphan medicinal products, vaccines and advanced therapy medicinal products” [9]. However, the HTA Coordination Group (CG) has not, as yet, released any specific methodologies for ATMPs. Instead, the generally applicable *Methodological and Practical Guidelines for Quantitative Evidence Synthesis: Direct and Indirect Comparisons*—published in March 2024—emphasise traditional evidence hierarchies that rely on RCTs with large sample sizes to detect small treatment effects in relatively short time periods. Such methods are often rooted in 20th century practises developed to assess and appraise conventional medicines, and struggle to accommodate 21st century technologies like ATMPs.

In response to these guidelines, ARM—along with an informal alliance of concerned stakeholders from the ATMP sector, including Cancer Patients Europe, Fondazione Telethon, and over 30 leading patient organisations, foundations and medical centres and societies [12]—expressed our concern that the methods adopted could deem the datasets on which most ATMPs are authorised as too unreliable and uncertain. By discrediting key evidence and restricting the possibilities for taking that evidence into account at the national level, the JCA risks introducing a new hurdle to patient access rather than streamlining the HTA process in Europe. In our Call to Action, we called on the HTA CG to adopt a more pragmatic and fit-for-purpose approach [12].

We welcome the HTA CG’s June 2024 clarification that “new approaches to evidence generation can be used even if they are not specifically mentioned in the guidance and will be evaluated by the JCA assessors based on existing literature” [23]. Indeed, real-world data, including data from well-designed patient registries, and retrospective and prospective observational studies, can and should be used to provide context and comparison for single-arm trials. These tools can be augmented where available by control arm data from prior RCTs. While these signals are encouraging, the March 2024 guidelines remain in effect, and we remain concerned about their practical applications.

At the same time, we also echo the more recent concerns expressed by the EAA Faculty in their November 2024 Open Letter on Methodological Guidances to the HTA CG and the European Commission. As stated by the EAA’s faculty, “addressing uncertainty in clinical development was identified as a key prerequisite for EU HTA to provide an additional benefit over the current standard of national assessments … Considering the current status of adopted methodological guidance documents, the evidence base for the currently adopted EU HTA methodological framework does not reach beyond an informal consensus among the involved members of the CG. Major uncertainty regarding the applicability, robustness and validity of those methods prevails” [24].

We hope that going forward, the HTA CG will develop additional practical guidance on non-RCT-based-evidence approaches, including guidance on the feasibility of RCTs and—when RCTs are not possible—practical guidance on evidence development approaches to fill data gaps, evidence expectations and requirements for collecting and processing non-randomised evidence (NRE), and the best methods and approaches for collecting and processing NRE (including RWE).

The collection of RWE has long been known to address the uncertainty around the durability of ATMPs in the long term. However, clear, consistent guidelines, and the state-of-the-art and preferred methods for conducting RWE generation are not available, and RWE has not yet been accepted or integrated into the assessment processes of all HTA bodies. At the same, those HTA bodies that do accept RWE have different requirements and approaches to comparisons and preferred HRQoL measures. This issue is not unique to ATMPs, but exists in many different rare diseases with a high unmet need. Despite an increasing awareness and interest in RWE, there is still a lack of harmonisation between countries in their approach and methods for collecting evidence. While HTDs must provide a clear rationale to justify their trial design and be transparent in data collection and methodology for their evidence package [25], emerging industry best practises in the use of RWE are in fact shaped by HTA guidelines. As such, persistent differences in country-specific guidelines can cause difficulties for trial sponsors.

The HTA CG has the opportunity to contribute to a robust framework for RWE that could increase its acceptance, harmonise its collection, and ensure complete and high-quality datasets. Indeed, the HTA CG could pragmatically evaluate and assess when external controls and in-market data collection are appropriate to allow patient access while additional data are generated to confirm value. This could in turn increase manufacturer confidence that investment in new treatment evidence is not a high risk and increasing the HTA CG’s and national HTA bodies’ acceptance of evidence due to greater clarity and transparency in methodologies [25].

## 5. Looking Ahead to the First JCAs for ATMPs

We believe that ATMPs are the present and future of medicines, driving innovation in health for years to come. The HTA Regulation and the JCA represent a generational opportunity to streamline decision-making and cement Europe’s position as a pioneer in innovative and flexible HTA approaches. As the JCA gets underway for ATMPs in January 2025, close monitoring of the first JCAs and first JCA reports—expected in 2026—and how they are used by Member States is essential. For example, if Member States request additional PICOs beyond those defined in the JCA, this could result in added burdens for manufacturers, particularly smaller companies, as JCA dossiers might supplement rather than replace country-specific submissions. This could risk diverting resources into markets where reimbursement may not ultimately be viable, underscoring the need for harmonised and predictable processes.

The lessons learned during the initial years of the JCA will lay the groundwork for its evolution. The European Commission’s planned review of the Regulation is expected to begin in 2027 and will provide an opportunity to refine the system and ensure it lives up to its potential. For developers launching ATMPs in 2025–2026, however, the reality is this may come too late.

Joint Scientific Consultations (JSCs) between HTDs and the HTA CG could help mitigate some of these issues. Article 16 of the Regulation states that JSCs shall “facilitate the generation of evidence that meets the likely evidence requirements of a subsequent JCA on that health technology”. This is essential for medicines at the cutting edge of innovation, like ATMPs, that face specific challenges with evidence generation. As the JCA does not foresee the possibility of JCA scoping meetings except in exceptional circumstances, it is crucial that HTDs have the possibility to discuss clinical study design aspects, including how to fill evidentiary gaps, with the HTA CG at an early stage. Unfortunately, the HTA CG draft work programme for 2025 only plans for between five and seven JSCs for pharmaceuticals against 25 JCAs. We believe a greater number of JSCs should be planned for, necessitating a higher level of resources and capacity in order to, ideally, ensure all demands for JSCs are satisfied.

At the same time, the HTA CG can, through the adoption of additional methodological guidance, develop EU-wide standards for Europe’s future approach to evidence-based medicine. As many European HTA bodies already adopt pragmatic approaches to assessments [26,27], either through separate pathways or by applying special considerations as part of the overall appraisal, an adapted HTA approach at the EU level would cast already existing practises into a formal framework. The methodologies and approaches will impact the patient access landscape across Europe for a generation. The JCA is an opportunity to improve the HTA Framework in Europe, particularly with regard to ATMPs. Transparent and consistent methodologies and approaches across the European Union could serve to reduce the inequalities in access to ATMPs, lead to greater clarity on evidentiary expectations for the developer, and clear plans for handling remaining evidentiary uncertainties.

The new HTA Regulation has already served to bring the EU HTA ecosystem closer together throughout Europe. Numerous benefits have already emerged from EU-wide collaboration through the implementation of the HTA Regulation, and will continue to emerge through joint work on the Joint Clinical Assessments. Specifically, a ‘levelling up’ effect can be observed, as cooperation encourages less experienced HTA bodies to adopt more advanced practises [28]—mirroring the evolution in the EMA’s CHMP process, where initial leadership from a few countries gradually broadened to a more equitable distribution of responsibilities across Member States. The standardisation and ‘portability’ of data, particularly in the context of RWE, will be critical for pooling evidence across Member States and ensuring robust, EU-wide analyses, especially for rare and very rare diseases. This highlights the JCA’s potential to drive widespread improvements and increasing expertise across Europe’s HTA landscape.

## 6. Conclusions—Recommendations Going Forward

Ensuring the success of the JCA system for ATMPs from the start will require that JCA assessors follow the HTA CG’s more pragmatic approach and exercise flexibility in assessing all types of available evidence for ATMPs, including single-arm trials, and accept RWE to help fill evidence gaps.

Ideally, additional resources and capacity should be made available to ensure that all demands for Joint Scientific Consultations (JSCs) can be satisfied.

National HTA agencies must also actively collaborate, embrace flexibility, and adopt pragmatic approaches to address uncertainties in JCA reports, develop methodologically innovative national guidance, and ensure open, transparent processes that prioritise patient access and do not create unnecessary barriers.

Realising the potential of the JCA will require continued close collaboration among all stakeholders, and the wider HTA ecosystem must continue to promote and develop methodological innovation around NRE outside the formal CG guidance.

By getting it right in the coming years, we can ensure that access to ATMPs becomes a reality for patients across Europe, and secure Europe’s position as a global leader in health innovation and assessment.
Key RecommendationsJCA assessors should adopt a pragmatic approach and exercise flexibility in assessing all types of available evidence for ATMPs.Additional resources and capacity should be made available to ensure all demands for JSCs can be satisfied.National HTA agencies must also actively collaborate, embrace flexibility, and adopt pragmatic approaches to address uncertainties in JCA reports.The wider HTA ecosystem must continue to promote and develop methodological innovation.

## Data Availability

The original data presented in the study are openly available at https://alliancerm.org/data/ (accessed on 21 March 2025) and https://alliancerm.org/sector-snapshot-august-2024/ (accessed on 21 March 2025).
